# Font Size Matters—Emotion and Attention in Cortical Responses to Written Words

**DOI:** 10.1371/journal.pone.0036042

**Published:** 2012-05-09

**Authors:** Mareike Bayer, Werner Sommer, Annekathrin Schacht

**Affiliations:** 1 Department of Psychology, Humboldt-Universität zu Berlin, Berlin, Germany; 2 Courant Research Centre Text Structures, University of Göttingen, Göttingen, Germany; Tel Aviv University, Israel

## Abstract

For emotional pictures with fear-, disgust-, or sex-related contents, stimulus size has been shown to increase emotion effects in attention-related event-related potentials (ERPs), presumably reflecting the enhanced biological impact of larger emotion-inducing pictures. If this is true, size should not enhance emotion effects for written words with symbolic and acquired meaning. Here, we investigated ERP effects of font size for emotional and neutral words. While P1 and N1 amplitudes were not affected by emotion, the early posterior negativity started earlier and lasted longer for large relative to small words. These results suggest that emotion-driven facilitation of attention is not necessarily based on biological relevance, but might generalize to stimuli with arbitrary perceptual features. This finding points to the high relevance of written language in today's society as an important source of emotional meaning.

## Introduction

In a complex environment where stimuli compete for attention, emotional stimuli such as sex-related pictures or threatening faces enjoy a processing advantage. It has been suggested that these emotional stimuli attract ‘motivated attention’ [1] because of their intrinsic biological relevance. This facilitation is evident in event-related potentials (ERPs) as enhanced negativity over occipital scalp sites for emotional compared to neutral stimuli, starting approximately 150 ms after stimulus onset [2]. The assumption that this early posterior negativity (EPN) reflects *intrinsic* bottom-up attention allocation is based on its temporal and topographical similarities to an ERP component triggered by *voluntary* attention allocation: When a subject's attention is directed to non-spatial stimulus features (e.g., color, shape), a so-called selection negativity is elicited over the posterior visual cortex [3]. While voluntary, top-down attentional modulations are mediated through a frontoparietal network, the transient facilitation of emotional stimuli seems to be modulated by the amygdala through reciprocal projections to the extrastriate visual cortex [4]. Furthermore, emotional stimuli also seem to activate the frontoparietal attention network [5], providing further evidence for the link between emotion and attention.

At a later processing stage, starting around 300 ms after stimulus onset, emotional stimuli elicit a long-lasting positivity over the centroparietal cortex (termed late positive complex, LPC), which presumably reflects higher-order stimulus evaluation [6]. Similar to the EPN, this ‘emotion component’ has a counterpart in a large body of emotion-unrelated research about the P300 component; augmented centroparietal P300 components are typically elicited by explicitly attended, task-relevant stimuli [7]. Taken together, the characteristics of emotion effects on the ERP suggest the involvement of attentional mechanisms at both early and late processing stages to underlie emotion facilitation.

Assuming that bottom-up attention allocation is based on the biological relevance of emotional stimuli, it is noteworthy that emotion effects are not only elicited by emotional pictures and facial expressions, but also by written verbal stimuli. Unlike pictorial stimuli, the processing of emotional words cannot rely on direct biological preparedness, cf. [8], but requires the translation of abstract symbols into meaning. Nonetheless, emotional words elicit comparable emotion effects in form of EPN and LPC, e.g., [9]–[11].

Given the links between emotion and attention outlined above, an important question concerns the interplay of both variables. How do variations of attention impact emotional processing? The dual nature of attention enables two distinct trigger mechanisms, namely voluntary, top-down attention and stimulus-driven, bottom-up attention.

In order to manipulate explicit attention to emotional stimuli, Schupp and colleagues [12] employed a counting paradigm. Within separate runs of the experiment, participants had to count the number of positive, neutral, or negative pictures, so that each emotional condition was presented as attended category and as non-attended category. This combination of top-down attention with stimulus-driven ‘emotional’ attention elicited additive effects on ERPs during sensory encoding, resulting in independent, though topographically similar, early negativities for emotion and explicit attention. In contrast, an interaction of emotion and attention was found for the LPC, where explicit attention increased the effects of emotion. Similar results have been reported for the EPN in response to emotional words. In a counting paradigm, emotional words elicited an EPN irrespective of whether they belonged to an attended or a non-attended word category [13].

In order to manipulate stimulus-driven, bottom-up attention, Codispoti and De Cesarei [14] presented emotional and neutral pictures in different stimulus sizes. An increase in picture size elicited increased amplitudes of skin-conductance responses as well as larger emotion-related difference in arousal ratings. Interestingly, a similar interaction of emotion and size was found for the EPN in the ERP, which started earlier and was more pronounced for large than for small pictures [15]. Assuming that the size of a picture reflects the distance to an object in real life, the interactive effect of emotion and attention on the EPN was suggested to result from the higher motivational relevance of apparently ‘closer’ objects. Notably, the interaction of emotion and stimulus size was limited to the EPN and was absent for the LPC, where the effects of emotion and size were additive. This is at variance with interactions of emotion and explicit attention mentioned above [12], which were present at higher-order stages, but not during sensory encoding.

The aim of the present study was to investigate the interplay of stimulus size and emotion effects for word stimuli. Assuming that the interaction of stimulus size and emotion reported for pictures results from higher biological relevance, a similar effect is unlikely to occur for written words: Since letters are entirely arbitrary, the concept of proximity – and thus of biological relevance – should not apply, and effects of emotion and stimulus size are likely to be additive. On the other hand, considering the profound impact of written language in our society, it seems conceivable to assume that written language acquired a *similar* form of relevance. Nowadays, written language is a major source of information and does not only convey facts and knowledge, but often carries high personal and social relevance. In this case, the potentiating effects of stimulus size may not be limited to biologically relevant stimuli, but may extend to learned symbolic stimuli. In order to investigate this question, we presented positive, neutral, and negative words in two different font sizes and measured event-related potentials while participants were reading the words.

## Methods

### Participants

Data was collected from twenty-five participants (mean age = 25.6 years, SD = 4.9; 18 women and 7 men). All were native German speakers, had normal or corrected-to-normal vision, and no neurological or psychiatric disorders according to self-report. Twenty-four participants were right-handed and one was left-handed according to the Edinburgh inventory [16]. Participation was reimbursed with course credit or 20 euros.

### Stimuli

Seventy-two German nouns were selected from the Berlin Affective Word List Reloaded [17], consisting of 24 high-arousing positive, 24 low-arousing neutral, and 24 high-arousing negative words. Stimulus categories differed significantly in their valence ratings, *F*(2,69) = 1403.45, *p*<.001; positive vs. neutral: *F*(1,46) = 770.25, *p*<.001; positive vs. negative: *F*(1,46) = 2639.03, *p*<.001; negative vs. neutral: *F*(1,46) = 704.15, *p*<.001. Positive and negative stimuli were matched for arousal, *F*(1,46) = 1.43, *p* = .71, but differed significantly from neutral words, *F*(2,69) = 82.51, *p*<.001; positive vs. neutral: *F*(1,46) = 100.86, *p*<.001; negative vs. neutral: *F*(1,46) = 264.58, *p*<.001. In addition, stimulus categories were controlled with respect to word frequency [18], number of letters and syllables, and imageability ratings, all *Fs*(2,69)<1. Stimulus characteristics are summarized in Table 1.

**Table 1:** Descriptive statistics (Means and Standard Deviations) of stimulus words.

	Valence	Arousal	Imageability	Word Length	Word Length	Frequency
	(−3–3)	(1–5)	(1–7)	(Number of Letters)	(Number of Syllables)	(Ftot/1mil)
**Positive**	2.1 (0.2)	3.3 (0.7)	5.5 (0.8)	6.3 (2.0)	2.0 (0.8)	27.7 (31.9)
**Neutral**	0.3 (0.3)	1.9 (0.2)	5.6 (0.4)	6.3 (1.2)	2.0 (0.8)	24.6 (29.2)
**Negative**	−2.1 (0.3)	3.5 (0.5)	5.5 (0.6)	6.4 (2.1)	2.1 (1.0)	24.8 (20.5)

### Procedure

The study has been approved by the ethics committee of the Department of Psychology at the University of Göttingen, Germany, and was conducted according to the Declaration of Helsinki. Upon arrival, participants were acquainted with the experimental procedure and signed informed consent. During the experiment, they were seated in a dimly lit, sound-attenuated chamber facing a computer screen positioned at a distance of 60 cm. All words were presented in two font sizes (*small*: 28 points, *large*: 125 points, Arial font) within consecutive blocks. Within each block, stimuli were randomized and presented twice; the order of blocks (*small* and *large*) was counterbalanced. Participants were instructed to attentively read the words. In order to ensure that subjects were paying attention, a 1-back task was randomly interspersed after 3 to 16 trials by placing a green frame around the presented word. Participants had to decide by button press whether the presented word was identical to the immediately preceding word or not. Stimuli were presented in light gray letters on a dark gray background, spanning a mean visual angle of 2.4×0.9° (small words) and 10.6×3.2° (large words). At the start of each trial, a letter mask was presented for 1 s, followed by the stimulus word for 3 s.

### EEG recording

The electroencephalogram was recorded from 61 electrodes referenced to the left mastoid. Vertical and horizontal electrooculograms were measured from four electrodes at the outer canthi and below both eyes. Electrode impedance was kept below 5 kΩ; signals were recorded with a sampling rate of 500 Hz and amplified with a bandpass filter of 0.032–70 Hz. Offline, data was average-referenced and corrected for blinks and eye movements using Surrogate Multiple Source Eye Correction [19]. The continuous EEG signal was then segmented into epochs of 1100 ms starting 100 ms before stimulus onset, and referred to a 100 ms pre-stimulus baseline. After epochs containing artifacts were discarded, ERP segments were averaged per subject and experimental condition.

### Data analysis

In order to investigate the time course of ERP modulations by emotional content and stimulus size, repeated-measures ANOVAS with the factors emotion (positive, neutral, negative) and electrode (58) were calculated in consecutive 10-ms time windows from 200 to 600 ms after stimulus onset, separately for small and large words. To prevent spurious results, only time windows with significant activations in at least three consecutive intervals were considered for subsequent analyses. This data-driven procedure was chosen since previous studies showed considerable temporal variance in the time course of emotion effects, e.g., [10], [11]. Furthermore, no study so far sought to investigate emotion effects to words that were presented at different font sizes; therefore, we could not derive a priori hypotheses regarding the time windows for this comparison. In a second step, mean activations in the time windows determined by the consecutive analyses were analyzed at specific regions of interest. In line with previous reports [9], [11], we selected a group of posterior electrodes for the EPN (PO9, PO7, PO8, PO10, P9, P7, P8, P10), and a second centroparietal group for the LPC (C3, C4, CP5, CP1, CP2, CP6, P5, P3, P4, P6), where both components showed their local maxima. Repeated measures ANOVAs included the factors emotion (positive, neutral, negative), size (large, small), hemisphere (left, right), and electrode (4/5, respectively).

In addition to EPN and LPC effects, we investigated whether emotional content also affected the amplitudes of the visually evoked components P1 and N1. To this aim, we analyzed mean amplitudes between 90 and 120 ms (P1) and between 150 and 170 ms (N1) with repeated-measures ANOVAs including the factors size (2), emotion (3) and electrode (58).

For analyses of scalp distributions of ERP effects of emotion and stimulus size we employed profile analyses as described by McCarthy and Wood [20]. First, differences in overall amplitude were eliminated by normalization of difference waves by the voltage range across electrodes within each participant and condition. In a second step, normalized difference waves were compared using repeated measures ANOVAs. Within these analyses, significant interactions of electrode and experimental factor (e.g., size) indicate topographical differences of ERP effects irrespective of overall ERP activity.

In all ANOVAs, Huynh-Feldt correction was applied to the degrees of freedom in order to correct for violations of the sphericity assumption. Note that all results will be reported with uncorrected degrees of freedom, but corrected *p*-values. Within all post-hoc comparisons, *p*-values were Bonferroni adjusted.

## Results

### P1 and N1

P1 amplitudes were enhanced for large compared to small words, *F*(57,1368) = 8.25, *p*<.001, *η^2^_p_* = .256. Furthermore, large words elicited less negative N1 amplitudes than small words, *F*(57,1368) = 5.26, *p*<.001, *η^2^_p_* = .180. Neither P1 nor N1 amplitudes were affected by the factor emotion, *F*s(114,2736)<1.

### Early posterior negativity

**Figure 1:**
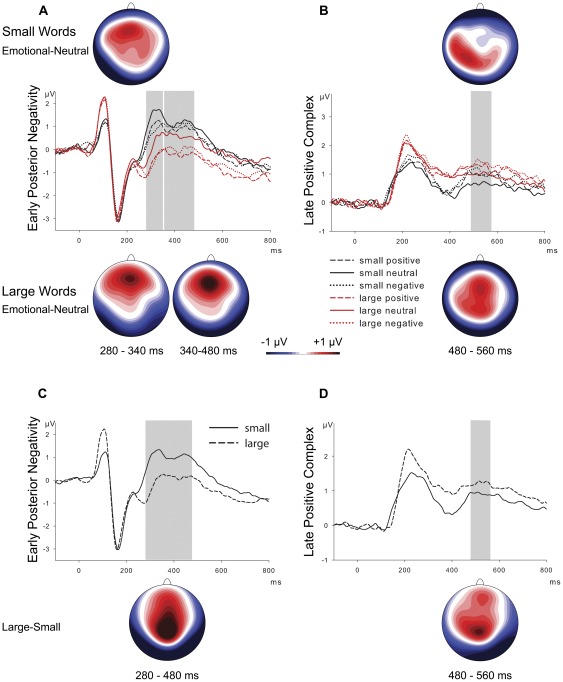
ERP effects of emotion and size. (A) Grand mean ERP waveforms for positive, neutral, and negative words of small and large size, collapsed over posterior EPN (ROI) electrodes. Scalp distributions show differences between emotional (positive and negative) and neutral words at indicated time intervals. (B) Grand means at centroparietal LPC electrodes and topographies of the late posterior complex as difference between emotional and neutral words in the indicated time range. (C) Effects of stimulus size on grand means over posterior EPN electrodes and scalp distribution of difference waves between large and small words in the time interval of the early posterior negativity. (D) Grand means for small and large words at centroparietal LPC electrodes and scalp distributions of difference ERPs between large and small words in the interval of the late positive complex.

Consecutive ANOVAs over all electrodes revealed emotion effects starting at 280 ms after stimulus onset for large words and at 290 ms for small words, respectively. While the emotion effect for small words ceased at 340 ms, the effect for large words lasted until the start of the late positivity at 480 ms after stimulus onset. Accordingly, ROI analyses at posterior electrodes were performed for the two time intervals from 280 to 340 and from 340 to 480 ms. Results of these analyses revealed a main effect of emotion in the initial interval of the EPN (280–340 ms), *F*(2,48) = 15.60, *p*<.001, *η^2^_p_* = .394. As presented in Figure 1A, both positive and negative words elicited enhanced negative amplitudes at posterior electrode sites relative to neutral words, *F*s(1,24)>19.12, *ps*<.001, *η^2^_p_*s>.443, but did not differ from each other, *F*(1,24) = 1.91, *p* = .54. The effect of stimulus size was evident in larger amplitudes of a posterior negativity for large stimuli, *F*(1,24) = 36.76, *p*<.001, *η^2^_p_* = .605 (see Fig. 1C).

Analyses of scalp distributions of the effects of stimulus size (large minus small) and emotion (emotional minus neutral, within both sizes) revealed no significant differences, all *F*s(57,1368)<1.75, *p*s>.113.

**Figure 2:**
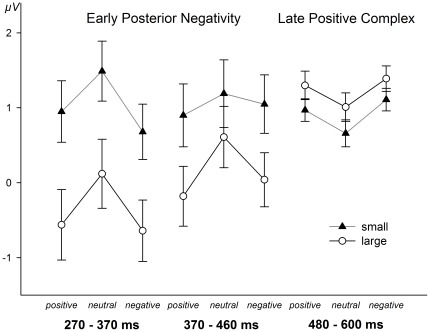
Mean EPN and LPC amplitudes. Mean amplitudes and Standard Errors of EPN and LPC separately for each emotion category and size conditions.

In the following interval from 340 to 480 ms, ANOVAs of mean amplitudes revealed a main effect of emotion, *F*(2,48) = 7.45, *p*<.01, *η^2^_p_* = .237, as well as an Emotion×Size interaction, *F*(2,48) = 3.49, *p*<.05, *η^2^_p_* = .127. This interaction was due to the fact that in this interval emotion effects were present for large words, *F*(2,48) = 9.48, *p*<.001, *η^2^_p_* = .283, but were not detectable in small words, *F*(2,48) = 1.59, *p* = .432. Large positive and negative words elicited enhanced EPN amplitudes as compared to large neutral words, *Fs*(1,24)>12.49, *ps*<.01, *η^2^_p_*s>.342, respectively, but again did not differ from each other, *F*(1,24)<1. As in the previous interval, analyses of scalp distributions of the emotion effect for large words (emotion minus neutral) and the effect of stimulus size (large minus small) did not reveal significant differences, *F*(57,1368) = 1.68, *p* = .153. Taken together, our analysis revealed that the EPN showed an earlier onset and lasted longer for large than for small words (see Fig. 1A and Fig. 2).

### Late positive complex

Exploratory ANOVAs revealed significant effects of emotion, ranging from 480 until 520 ms after stimulus onset for small words and until 560 ms for large words, respectively. Consequently, for the comparison of emotion effects at the LPC region of interest, we analyzed mean amplitudes in the time windows from 480 to 520 ms and from 520 to 560 ms. In both time intervals, analyses revealed significant main effects of emotion, *F*(2,48) = 8.68, *p*<.01, *η^2^_p_* = .265 and *F*(2,48) = 6.33, *p*<.01, *η^2^_p_* = .209, respectively, that were due to larger amplitudes of a centroparietal positivity for negative compared to neutral words, *F*s(1,24)>7.31, *p*s<.05, *η^2^_p_*s>.234, and for positive compared to neutral words, *F*s(1,24)>7.99, *p*s<.05, *η^2^_p_*s>.250, whereas positive and negative words did not differ from each other, *F*s(1,24)<1.64, *p*s>.231; results are depicted in Figure 1B. Furthermore, ANOVAs revealed an effect of stimulus size in both time intervals, *Fs*(1,24)>7.89, *ps*<.05, *η^2^_p_*s>.247, due to enhanced amplitudes at centroparietal electrodes for large compared to small words (see Fig. 1C). However, unlike for the later EPN interval, there were no significant interactions between stimulus size and emotion in both LPC intervals, indicating comparable emotion effects at ROI electrodes for small and large words in the time interval from 480 to 560 ms after stimulus onset.

Analyses of scalp distributions revealed no significant differences between the emotion effects for large and small words in both time intervals of the LPC, *F*s(59, 1357)<1.14, *p*s>.340. Furthermore, both topographies did not statistically differ from the effects of stimulus size in the respective time window, all *F*s (59, 1357)<1.80, *p*s>.096.

## Discussion

The present study aimed to investigate the interplay of stimulus size and emotional content in written words. To this end, we presented words of positive, negative, and neutral valence in two font sizes while recording ERPs. The processing of emotional relative to neutral words elicited emotion effects in ERPs in the form of an EPN and a LPC. Furthermore, an interaction between size and emotion was apparent within the interval of the EPN. For large words, it showed an earlier onset and a longer duration than for small words.

Despite the symbolic nature of word recognition, evidence from ERPs and neuroimaging data suggested that similar brain structures, most importantly the amygdala, are involved in the detection and facilitation of emotional information across stimulus domains [21], [22]. Beyond these structural similarities, our results showed a functional similarity across stimulus domains: An increase in stimulus size caused augmented emotion effects during sensory encoding, replicating effects of stimulus size for emotional pictures [15]. In the following, we will discuss three important implications of these results.

First, in real life, the distance from an object influences its biological relevance for the organism [23]. Aggressors, for example, appear to be more dangerous the closer they get to the individual. As pictures size corresponds to the proximity of an object in reality, the increase of emotion effects during sensory encoding in ERPs was supposed to result from the increased stimulus relevance of larger pictures [15]. However, such a reference to biological relevance and real-life distance cannot be applied in the case of words, since written language is not an image of reality, but a symbolic system. Nonetheless, variation of font size elicited similar modulations of the EPN as variations of pictures size, showing increased emotion effects for larger stimuli. This provides first evidence that the mechanism responsible for interactions of emotional content and stimulus-triggered attention is not limited to biologically relevant stimuli, but is also engaged in the processing of symbolic stimuli. This finding suggests that instead of *biological* relevance alone, a more *general* form of stimulus relevance – as for example stimulus size – may play a causal role in the interaction of size and emotional content. Furthermore, it seems plausible to suggest that the mechanism of sensory facilitation was originally based on a biological, survival-relevant form of relevance, but has then generalized to written words, probably reflecting today's high social relevance of language.

A second important finding concerns the fact that interactions of emotion and stimulus size were limited to sensory encoding, while effects at higher-order processing stages showed an additive relationship. The same pattern of results was reported for size manipulations of emotional pictures [15]. These findings are in contrast to manipulations of explicit, top-down attention, which does not influence the EPN, but increases the LPC [12], [13]. Taken together, these results suggest that stimulus-triggered attention interacts with emotional processing at the stage of sensory encoding, while top-down attention seems to influence emotion effects during higher-order stimulus evaluation. Furthermore, these interactions seem to occur irrespective of stimulus category, supporting the notion of a general system for emotion detection across stimulus domains.

A final remark concerns the time course of emotion effects, i.e., their functional locus in word processing. In the present study, the factor emotion did not affect the stage of early perceptual processing, which is reflected in the visually evoked components P1 and N1. Both components were modulated by stimulus size, caused by their sensitivity to physical stimulus features. Following this stage of initial perceptual processing, interactions between size and emotion were visible in the EPN. Originally described for emotional pictures, this component was suggested to indicate enhanced stimulus-triggered attention allocation to sensory processing of emotional as compared to neutral stimuli [2], presumably resulting from feedback from the amygdala to the extrastriate cortex [4]. In the case of visually presented words, the EPN occurs at a lexico-semantic processing stage, i.e., starting only with the access to the mental lexicon or shortly thereafter [11], [24]. Consequently, the EPN to emotional words has often been reported at longer latencies as compared to affective pictures or emotional facial expressions [2], [10]. Considering its lexico-semantic locus, one might argue that the attention allocation reflected by the EPN to words might not be purely stimulus-triggered in nature, as it had been suggested for pictures. A recent debate on the automaticity of the EPN refers to a similar aspect: On the one hand, a number of studies suggested that the EPN was elicited automatically, i.e., irrespective of task demands [2]. On the other hand, this view was challenged by findings indicating that EPN modulations are indeed sensitive to task requirements [25], [26]. However, the fact that stimulus size and emotion show an interactive influence on the EPN in the present study implies that the EPN, although probably also influenced by top-down attention, to some extend reflects stimulus-triggered attention allocation.

In conclusion, the present results show that large font size leads to an increase of early emotion effects in ERPs for written words. These findings indicate that interactions of emotion and stimulus size are not limited to biologically relevant objects, but might also be triggered by symbolic stimuli, pointing towards the high relevance of written language as a source of emotional meaning. The power of large font size to enhance emotion effects may, for instance, be one reason why headlines written in big letters are popular and evidently successful in the yellow press media.
